# Targeting Rho GTPase Signaling Networks in Cancer

**DOI:** 10.3389/fcell.2020.00222

**Published:** 2020-04-03

**Authors:** Natasha S. Clayton, Anne J. Ridley

**Affiliations:** School of Cellular and Molecular Medicine, University of Bristol, Bristol, United Kingdom

**Keywords:** cancer, GTPase, invasion, metastasis, Rho

## Abstract

As key regulators of cytoskeletal dynamics, Rho GTPases coordinate a wide range of cellular processes, including cell polarity, cell migration, and cell cycle progression. The adoption of a pro-migratory phenotype enables cancer cells to invade the stroma surrounding the primary tumor and move toward and enter blood or lymphatic vessels. Targeting these early events could reduce the progression to metastatic disease, the leading cause of cancer-related deaths. Rho GTPases play a key role in the formation of dynamic actin-rich membrane protrusions and the turnover of cell-cell and cell-extracellular matrix adhesions required for efficient cancer cell invasion. Here, we discuss the roles of Rho GTPases in cancer, their validation as therapeutic targets and the challenges of developing clinically viable Rho GTPase inhibitors. We review other therapeutic targets in the wider Rho GTPase signaling network and focus on the four best characterized effector families: p21-activated kinases (PAKs), Rho-associated protein kinases (ROCKs), atypical protein kinase Cs (aPKCs), and myotonic dystrophy kinase-related Cdc42-binding kinases (MRCKs).

## Introduction

**FIGURE 1:**
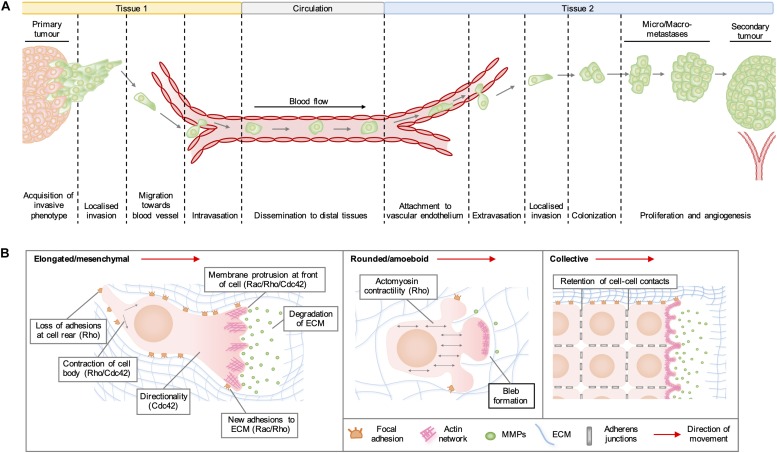
**(A)** Stages of cancer metastasis. A subset of cancer cells in the primary tumor acquire an invasive phenotype and spread into the surrounding stroma, either collectively or as single cells. Some invading cells migrate toward the tumor neovasculature and enter the blood stream by migrating through vascular endothelial cell junctions in a process known as intravasation. These cancer cells can be transported by the circulation to distal tissues, where they enter narrower vessels that permit their attachment to vascular endothelial cells. Following attachment, cancer cells commonly extravasate as single cells by migrating through endothelial cell junctions and then invade into the stroma of the secondary organ. These cells may form a metastatic niche if supported by survival and growth signals in the new micro-environment. Further cell proliferation will give rise to micro- and macro-metastases and a secondary tumor is established by the formation of a new blood supply through neo-angiogenesis. **(B)** Modes of cell migration. Elongated cell migration involves the extension of actin-rich protrusions at the front of the cell and the localized release of matrix metalloproteinases (MMPs), which degrade extracellular matrix (ECM) proteins and create space into which the cell can move. The formation of new adhesions at the front of the cell and contraction of the cell body pull the cell in the direction of movement, whilst loss of ECM adhesions at the rear allows the cell to migrate forward. During collective cell migration, neighboring cells within a tissue remain physically linked by adherens junctions. Cells at the invasive front extend actin-rich protrusions facing the direction of movement, which form new adhesions with the ECM and enable the generation of traction forces that pull neighboring cells forward. During rounded cell migration, high actomyosin contractility produces hydrostatic pressure, which leads to the formation of membrane blebs devoid of filamentous actin at the front of the migrating cell. Highly dynamic membrane blebs fill pre-existing spaces in the matrix and form only weak attachments to the ECM.

Rho GTPases are a family of highly conserved GTPases that are encoded by 20 genes in humans and regulate a range of cellular functions, including vesicular transport, gene expression, neuronal development, and cell division. By far the best characterized of Rho GTPase functions is the organization of the actin cytoskeleton into structures required for cell migration. In cancer, the adoption of a pro-migratory phenotype enables tumor cells to invade the stroma surrounding the primary tumor, migrate toward blood vessels and enter the circulation (Figure 1A). Migrating cells extend membrane protrusions at the leading edge, which are driven forward by localized actin polymerization (Figure 1B). In general, the formation of lamellipodia is regulated by Rac and Rho, whilst Cdc42 activity drives the formation of filopodia and maintains the cellular polarization required for directional migration. Rho regulates actomyosin contractility, which can lead to the formation of actin stress fibers and focal adhesions on rigid substrata, which are required to generate the traction forces needed to pull the cell body in the direction of movement. Migration of cells in 3D often requires the degradation of matrix components by proteases released from invadopodia, which are actin-rich structures regulated by RhoA, RhoC, and Cdc42 (Lawson and Ridley, 2018).

Altered expression of several Rho GTPases has been reported in a variety of human tumors (reviewed in Svensmark and Brakebusch, 2019). Whilst mutations in genes encoding Rho GTPases are rare in cancer, somatic mutations have been reported in *RHOA, RHOB, RAC1, RAC2*, and *CDC42* (Hurst et al., 2017; Aspenström, 2018). Here, we comment on the recent advances in targeting Rho GTPase activation and focus on inhibition of Rho GTPase effectors as a viable therapeutic strategy in cancer treatment.

## Targeting Rho Gtpase Activation

**FIGURE 2:**
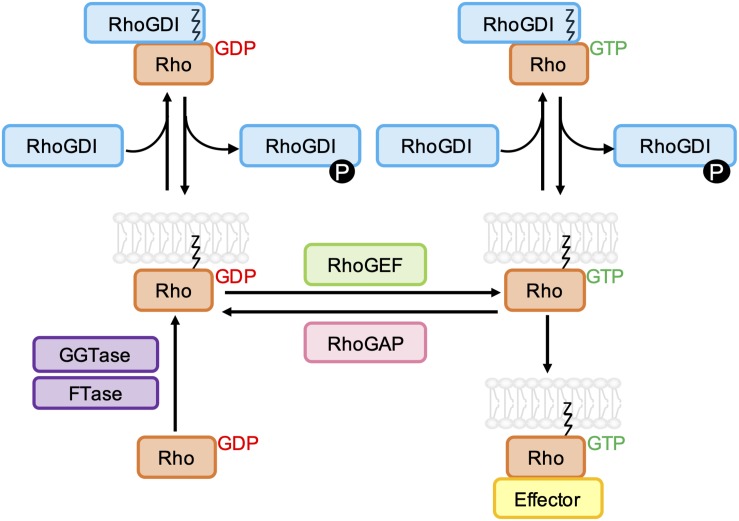
Schematic representation of the classical Rho GTPase regulatory cycle. GDP-bound Rho GTPases are prenylated at a C-terminal CAAX sequence by farnesyltransferase (FTase) and/or geranylgeranyltransferase type I (GGTase-I), which mediates their association with biological membranes. Rho guanine nucleotide exchange factors (RhoGEFs) promote the dissociation of GDP and uptake of GTP, which permits the interaction of Rho GTPases with effector proteins. GTPase-effector interactions are terminated by the hydrolysis of bound GTP to GDP, which is accelerated by Rho GTPase-activating proteins (RhoGAPs). Both GDP- and GTP-bound Rho GTPases can be negatively regulated by Rho guanine nucleotide dissociation inhibitors (RhoGDIs), which bind to and sequester the prenyl group, resulting in relocalization of the Rho GTPase to the cytoplasm. RhoGDI-Rho GTPase binding can be regulated by phosphorylation of RhoGDIs by kinases Src, PAK, and PKC.

The binding of GTP to the nucleotide binding pocket of membrane anchored Rho GTPases leads to a conformational change that stimulates their interaction with various downstream effectors, including protein kinases and scaffold/adaptor-like proteins. The activity of most Rho GTPases is terminated by the hydrolysis of bound GTP to GDP, and the dissociation of GDP is required before another GTP molecule can bind. For the classical Rho GTPases, this cycling between GTP- and GDP-bound states is tightly regulated by guanine nucleotide exchange factors (GEFs) and GTPase-activating proteins (GAPs) (Figure 2). Rho GTPases can also be regulated by guanine nucleotide dissociation inhibitor (GDI) binding, which sequesters Rho GTPases in the cytoplasm (Golding et al., 2019).

Significant effort has been made to develop compounds that modulate multiple stages of the GDP/GTP cycle (Jansen et al., 2018; Maldonado and Dharmawardhane, 2018). These include compounds that inhibit GEF-GTPase binding, which have produced promising results in preclinical cancer models. For example, EHop-016 blocks the interaction of Rac1 with Vav2 (Montalvo-Ortiz et al., 2012), and has been shown to enhance the anti-tumor effect of cisplatin in xenograft models of esophageal squamous cell carcinoma (Zeng et al., 2019). ZCL278 blocks the interaction of Cdc42 with intersectin (Friesland et al., 2013) and can inhibit the growth of lung cancer xenografts (Aguilar et al., 2019). It is important to note that the activity of atypical Rho GTPases is unlikely to be regulated by GEF/GAP interactions (Aspenstrom et al., 2007; Hodge and Ridley, 2017), and therefore alternative strategies will be required to target these family members effectively.

In contrast to the prevailing view that the GDP-bound forms of Rho GTPases are inactive, recent data have identified a role for Rho-GDP in oncogenic signaling. The RhoA driver mutation G17V, which disrupts GTP binding, is frequently identified in T-cell lymphoma (Yoo et al., 2014) and studies in *Dictyostelium* cells demonstrate that phosphorylation of RhoA-GDP at S192 leads to increased mTORC2 activity and phosphorylation of AKT (Senoo et al., 2019). These findings suggest that, in certain cancers, targeting GTP-bound Rho GTPases may offer little therapeutic benefit.

Membrane localization of Rho GTPases following post-translational C-terminal lipid modifications, including prenylation and palmitoylation, is crucial for their function (Mitin et al., 2012). The therapeutic potential of prenylation inhibitors is currently being investigated. The geranylgeranyltransferase type I inhibitor GGTI-2418 (PTX-100) has shown anti-tumor effects in breast cancer xenograft models (Aspenstrom et al., 2007) and was well tolerated in an initial phase I clinical trial (Karasic et al., 2019), although further phase I trials are required to optimize dosing and assess responses in patients with advanced malignancies (NCT03900442). Rho GTPase membrane localization can also be inhibited by treatment with statins, which suppress cholesterol biosynthesis, reducing the abundance of farnesyl pyrophosphate and geranylgeranyl pyrophosphate (Denoyelle et al., 2001). Statins are widely prescribed for treatment of cardiovascular disease (Tobert, 2003) and have been reported to reduce cancer recurrence and mortality in several cancer types (Beckwitt et al., 2018). However, this likely reflects the modulation of multiple pathways by statins, including inhibition of Rho GTPases.

A more promising approach to targeting Rho GTPase signaling is through the direct inhibition of Rho GTPase effectors. Here, we discuss the roles of PAKs, ROCKs, MRCKs, and aPKCs in Rho GTPase signaling and review recent advances in targeting these proteins in cancer.

## P21-Associated Kinases (PAKS)

**FIGURE 3:**
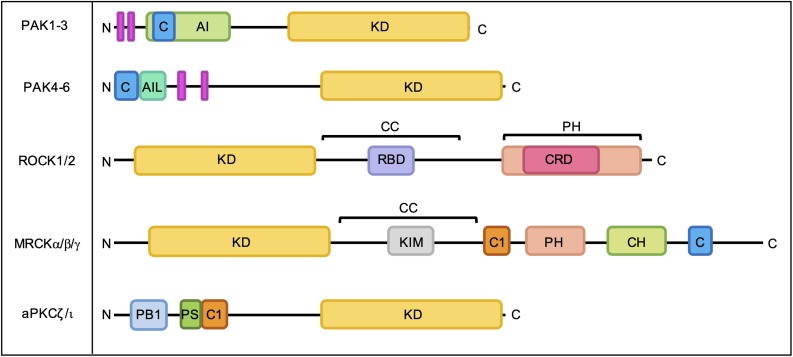
Domain organization of PAKs, ROCKs, MRCKs, and aPKCs. C, Cdc42- and Rac-Interactive Binding (CRIB) domain; AI, autoinhibitory domain; KD, kinase domain; AIL, autoinhibitory-like domain; RBD, Rho-binding domain; CC, coiled-coil region; PH, pleckstrin homology domain; CRD, cysteine-rich domain; C1, C1 domain; CH, citron homology domain; PB1, Phox Bem1 domain; PS, pseudosubstrate motif. Proline-rich regions are indicated by pink bars.

**FIGURE 4:**
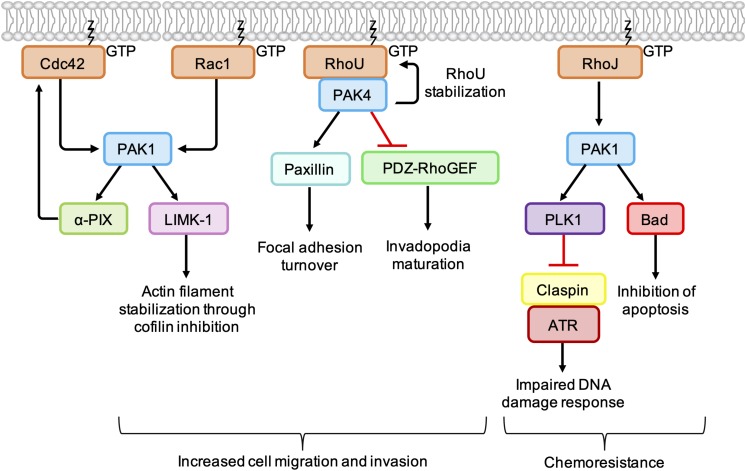
Cancer-associated PAK signaling pathways activated downstream of Rho GTPases. PAK1 phosphorylates LIM Kinase-1 (LIMK-1), which stabilizes actin filaments through inhibition of cofilin. PAK1 also binds to and stimulates the GEF α-PIX, leading to an increase in Cdc42 activation. PAK1 activates PLK1, which leads to phosphorylation and degradation of claspin. In the absence of claspin, ATR is uncoupled from its effectors, resulting in an impaired DNA damage response. PAK1 also phosphorylates the pro-apoptotic protein Bad, which prevents its association with Bcl-2 and inhibits apoptosis. PAK4 binding protects RhoU from proteasomal degradation and promotes focal adhesion turnover by providing a scaffold that promotes the phosphorylation of paxillin. PAK4 also inhibits PDZ-RhoGEF, which suppresses the activation of RhoA and promotes invadopodia maturation.

The best characterized Rho GTPase effectors are the highly conserved p21-activated kinases (PAKs), which are found in almost all eukaryotes except plants (Hofmann et al., 2004). In humans, the PAKs comprise a family of six serine/threonine kinases which can be divided into two subgroups based on sequence homology and structural similarity. All PAKs possess an N-terminal Cdc42- and Rac-Interactive Binding (CRIB) domain, which mediates their interaction with GTP-bound Rho GTPases, and a conserved kinase domain at the C-terminus (Figure 3). Group I PAKs (PAK1-3) possess an autoinhibitory domain which interacts with the kinase domain in a cis-autoinhibitory interaction. GTPase binding disrupts this autoinhibition and leads to the dimerization and trans-autophosphorylation of two PAK monomers, resulting in their full activation (Sorrell et al., 2019). In contrast, group II PAKs (PAK4-6) show constitutive activation loop autophosphorylation, which is not affected by the binding of Rho GTPases. Group II PAKs are instead activated by the dissociation of a proline-rich psudosubstrate region from the kinase domain, which may be triggered by the binding of an SH3-domain containing protein (Ha et al., 2012). According to this model, Rho GTPases may promote the activation of group II PAKs by facilitating these protein-protein interactions. PAKs were initially identified as hits in a screen for Cdc42 and Rac1 binding partners (Manser et al., 1994) and have since been shown to interact with a number of other Rho GTPases. PAK signaling downstream of Rho GTPase activity contributes to multiple cellular responses including cell migration, cell survival and sensitivity to certain chemotherapeutic agents (Figure 4; Rane and Minden, 2019).

PAKs are involved in membrane protrusion and focal adhesion turnover by promoting the phosphorylation of proteins involved in actin dynamics (Figure 4). Following activation by Cdc42/Rac1, PAK1 phosphorylates LIM Kinase-1 (LIMK1), which in turn phosphorylates and inactivates cofilin, suppressing the disassembly of actin filaments at the leading edge. Interaction of Rac1 with the WAVE regulatory complex leads to activation of the actin nucleation complex Arp2/3, which when combined with localized inactivation of cofilin, leads to formation of the branched actin network characteristic of lamellipodia (Jansen et al., 2018). In breast cancer cell lines, PAK4 has been shown form a complex with RhoU, providing a scaffold leading to increased phosphorylation of paxillin at S272, a crucial step in focal adhesion turnover (Nayal et al., 2006; Dart et al., 2015).

PAKs also contribute to cell migration and invasion by influencing the spatiotemporal activation of Rho GTPases. PAK4 is known to directly bind and inhibit PDZ-RhoGEF (Barac et al., 2004), leading to an indirect inhibition of RhoA. In melanoma cell lines, depletion of PAK4 was associated with an increase in RhoA activity and a decrease in the percentage of cells with mature degradative invadopodia. Expression of PDZ-RhoGEF dominant negative mutants rescued invadopodia in PAK4-depleted melanoma cells, which suggests that a PAK4-mediated reduction in RhoA activity is required for invadopodium maturation. In the same cell lines, depletion of PAK1 was associated with a decrease in RhoA activity and fewer nascent invadopodial actin puncta, suggesting that PAK1 acts upstream of PAK4 to drive the early stages of invadopodium formation (Nicholas et al., 2016). This suggests that, in addition to the tight spatiotemporal control of RhoC activity (Bravo-Cordero et al., 2011), co-ordinated activation of RhoA at different stages of the invadopodium lifecycle is required for efficient cancer cell invasion.

Group I PAKs can bind directly to the GEFs α-PIX and β-PIX and influence their activity and/or localization (Manser et al., 1998). PAK1 binding has been shown to stimulate the GEF activity of α-PIX, resulting in an increase in GTP-bound Cdc42 (Daniels et al., 1999). Other work has shown that PAK1 and Rac1 compete for binding to β-PIX and that the PAK1-β-PIX interaction is negatively regulated by PAK1 autophosphorylation. This may provide a mechanism through which other Rho GTPases, such as Cdc42, influence the spatio-temporal activation of Rac1 (ten Klooster et al., 2006). However, PAK1 activation at the leading edge of fibroblasts has been shown to recruit β-PIX and promote Rac1 activation (Cau and Hall, 2005). Thus, the interplay between PAK1, β-PIX, and Rac1 is likely to be complex and context specific (Jansen et al., 2018).

PAK1 activation can contribute to chemoresistance in melanoma through the inhibition of pro-apoptotic signaling pathways. For example, RhoJ is overexpressed in metastatic melanoma (Ho et al., 2012) and treatment of melanoma cell lines with cisplatin was found to promote PAK1 autophosphorylation in a RhoJ-dependent manner (Figure 4). PAK1 activation led to an uncoupling of the kinase ATR from its effectors Chk1 and ATF2, which resulted in an impaired DNA damage response and reduced apoptosis. The observation that PAK1 depletion sensitized melanoma cell lines to cisplatin suggests that combination treatments of PAK1 inhibitors and DNA-damaging agents may improve chemoresponsiveness in certain human tumors (Ho et al., 2012). In addition, BRAF mutant melanoma cell lines, PAK1 has been shown to phosphorylate the pro-apoptotic protein Bad (Figure 4), preventing its association with Bcl-2 and the subsequent release of cytochrome C from mitochondria (Schurmann et al., 2000; Ruiz et al., 2017). Treatment with the PAK inhibitor FRAX597 led to a decrease in Bad phosphorylation and induced more apoptosis within 24 h in BRAF mutant RhoJ positive melanoma cell lines than the BRAF inhibitors Vemurafenib and Trametinb (Ruiz et al., 2017).

Several ATP-competitive PAK inhibitors have been developed, although specificity has proven challenging, due to the structural similarity of PAK catalytic domains, as well as the relative flexibility and “open” conformation of the ATP-binding cleft (Semenova and Chernoff, 2017). FRAX597 is an orally available group I PAK inhibitor which has been shown to inhibit cancer cell growth *in vitro* (Chow et al., 2012; Semenova et al., 2017; Tan et al., 2017; Araiza-Olivera et al., 2018) and to sensitize orthotopic tumors to gemcitabene in a murine model of pancreatic cancer (Yeo et al., 2016). The closely related inhibitor FRAX486 showed anti-proliferative effects in a mouse model of childhood acute lymphoblastic leukemia (Siekmann et al., 2018) and the analog FRAX1036 was recently found to inhibit tumor growth in murine models of breast (Korobeynikov et al., 2019) and thyroid (Knippler et al., 2019) cancer. However, FRAX1036 has been found to adversely inhibit hERG potassium channels, suggesting that FRAX1036 and its analogs may not suitable for clinical use (Ndubaku et al., 2015). Glaucarubinone, first developed as an antimalarial, was found to inhibit the activation of both PAK1 and PAK4, which may prove advantageous in treating cancers where the co-ordinated activity of these isoforms contributes to disease progression (Yeo et al., 2014; Nicholas et al., 2016). Glaucarubinone reduced the growth of pancreatic cancer xenografts, and glaucarubinone and gemcitabine were found to have a synergistic effect on the inhibition of PAK1/4 activation and tumor growth (Yeo et al., 2014). Although originally developed as a PAK4 inhibitor, the Pfizer compound PF-3758309 inhibits all PAK family kinases and is the only ATP-competitive PAK inhibitor to have reached clinical trials (Semenova and Chernoff, 2017). Following a phase I trial in patients with advanced solid tumors, PF-3758309 was withdrawn from clinical investigation due to poor bioavailability, adverse effects and lack of tumor response (Mileshkin et al., 2011).

KPT-9274 is an allosteric PAK4 inhibitor which binds to and destabilizes PAK4 (Rane et al., 2017). KPT-9274 and its analogs may therefore prove superior to kinase inhibitors in treating certain cancers as they could provide dual blockade of PAK4 kinase-dependent and independent functions. KPT-9274 has been shown to inhibit tumor growth of a number of pre-clinical cancer models (Rane and Minden, 2019) and is currently in phase I clinical trials for non-Hodgkin lymphoma and advanced solid malignancies (NCT02702492).

## Rho-Associated Protein Kinases (Rocks)

The Rho GTPase effectors ROCK1 and ROCK2 are AGC-family serine/threonine kinases that are involved in a diverse range of cellular processes, including cell motility, cell survival and proliferation, gene transcription, differentiation, and angiogenesis (Wei et al., 2016). Here, we focus on the role of ROCK in promoting actin polymerization and actomyosin contractility.

Both ROCKs have an N-terminal kinase domain, a coiled-coil region containing a Rho-binding domain (RBD) and a C-terminal pleckstrin homology (PH) domain (Figure 3; Morgan-Fisher et al., 2013). ROCKs exist as constitutive parallel homodimers (Truebestein et al., 2015) and interaction with membrane lipids (Yoneda et al., 2005), cleavage by granzyme B or caspases (Sebbagh et al., 2001; Sebbagh et al., 2005) and binding of RhoA, RhoB, and RhoC (Amano et al., 2000) have been shown to stimulate ROCK signaling. ROCK1 and ROCK2 share 92% sequence homology within their kinase domains (Nakagawa et al., 1996) and have many common substrates (Yoneda et al., 2005), yet are not functionally redundant (Jerrell et al., 2017).

**FIGURE 5:**
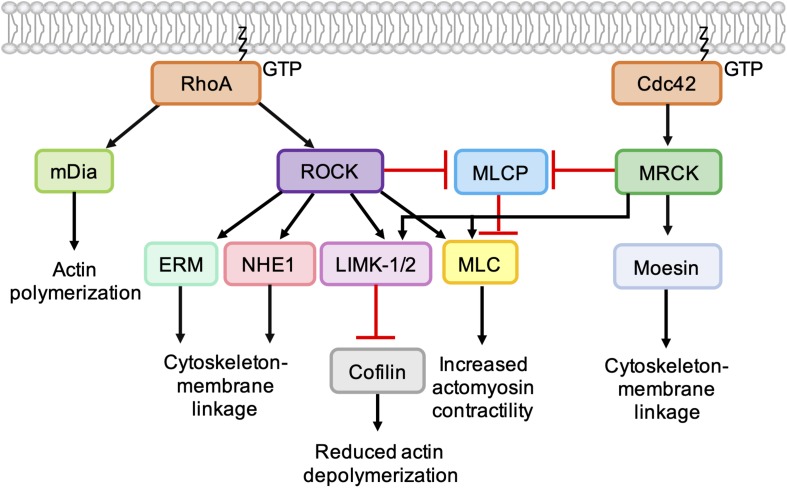
Role of Cdc42-MRCK signaling and RhoA-ROCK signaling in regulation of the actin cytoskeleton. RhoA directly binds and activates the formin mDia, which nucleates the formation of unbranched actin filaments. ROCK activation downstream of RhoA leads to phosphorylation of LIMK-1 and LIMK-2, which phosphorylate and inactivate cofilin, leading to a reduction in actin depolymerization. ROCK activation leads to an increase in actomyosin contractility via phosphorylation of myosin light chain (MLC) and inhibition of myosin light chain phosphatase (MLCP). ROCK also phosphorylates ERM proteins (ezrin, radixin, and moesin) and NHE1 (Na+/H+ -Exchanger 1) to enhance coupling of the actin cytoskeleton to integral membrane proteins. Like ROCK, MRCK activation leads to decreased actin depolymerization via phosphorylation LIMK-1 and LIMK-2 and increased actomyosin contractility via MLC phosphorylation. Phosphorylation of moesin by MRCKα may enhance coupling of the actin cytoskeleton to integral membrane proteins.

During cell migration, ROCK promotes the extension of actin-rich membrane protrusions through the activation of LIMK1/2 and inhibition of cofilin (Ohashi et al., 2000) and also stimulates cell body contraction through the generation of actomyosin contractility (Figure 5). Following activation by RhoA and RhoC, ROCK directly phosphorylates the myosin light chain (MLC) of non-muscle myosin II and inhibits MLC phosphatase through phosphorylation of the myosin binding subunit (Julian and Olson, 2014). Phosphorylation of MLC on Ser19 and Thr18 influences the ATPase activity of the myosin heavy chain (MHC) head groups, which move along actin filaments to produce contractile force (Mizutani et al., 2006). In a 3D environment, single cells have been shown to utilize two distinct modes of motility that are driven by different Rho signaling pathways (Figure 1B). Rac1 activity drives elongated cell motility, which is characterized by cell polarization, the extension of F-actin-rich protrusions and degradation of the ECM. In contrast, RhoA/C signaling through ROCK promotes a rounded bleb-based mode of motility driven by high levels of actomyosin contractility, which enables the cell to squeeze into pre-existing spaces and deform the ECM without the need for significant pericellular proteolysis (Wyckoff et al., 2006). To adopt a rounded mode of migration, Rho-ROCK signaling is often coupled to inhibition of Rac1 signaling, which can be achieved by stimulation of the Rac GAP ARHGAP22 downstream of ROCK activation. During elongated cell motility, active Rac1 suppresses the high levels of actomyosin contractility required for rounded motility via its effector WAVE2, which negatively regulates MLC phosphorylation (Sanz-Moreno et al., 2008). Cancer cells have been shown to convert between elongated and rounded modes of motility, suggesting that inhibition of both modes will be required to effectively prevent metastasis, for example via dual inhibition of secreted proteases and ROCK activity (Yamada and Sixt, 2019).

Deregulation of Rho-ROCK signaling has been identified in a number of human cancers and correlated with disease progression (Rodriguez-Hernandez et al., 2016). The *ROCK1* gene is amplified in a number of lung and gastric cancers as well as in head-and-neck squamous cell carcinoma (Svensmark and Brakebusch, 2019), whilst ROCK2 amplification has been associated with peripheral nerve sheath tumors (Upadhyaya et al., 2012). Three somatic mutations were identified in ROCK1 that result in activating C-terminal truncations (Lochhead et al., 2010) and polymorphisms in both ROCK1 and ROCK2 have been associated with colorectal cancer development (Sari et al., 2013).

Although significant effort has been made to develop ROCK inhibitors for cancer intervention, the vast majority of compounds have not progressed to clinical trials. The ATP-competitive ROCK inhibitors Y-27632 and fasudil have been used extensively as tool compounds and have been shown to inhibit cancer cell migration in various *in vitro* cancer models (Wei et al., 2016). Although fasudil is clinically approved for the acute treatment of cerebral vasospasm in Japan and China, its pharmacokinetic profile makes it unsuitable for use in chemotherapy (Rath et al., 2018). More recently developed pan-ROCK inhibitors, such as OXA-06, PT262, RKI-1447, and CTT129253, show more potent ROCK inhibition and achieve anti-tumor effects in pre-clinical models (reviewed in Wei et al., 2016; Jansen et al., 2018). Several compounds displaying selectivity for ROCK2 over ROCK1 have also been reported, although the efficacy of these compounds has mostly been tested in models of other diseases, such as glaucoma, hypertension and chronic kidney disease (Wei et al., 2016). To date, only one ROCK inhibitor has progressed into clinical trials for cancer treatment. AT13148 is an orally available multi-AGC kinase inhibitor that was identified through a fragment-based screen and was found to potently inhibit ROCK1 and ROCK2 (Yap et al., 2012). AT13148 was shown to have anti-tumor effects in pre-clinical models of pancreatic (Rath et al., 2018), breast, prostate and uterine cancer (Yap et al., 2012) and was well tolerated in a phase I clinical trial in patients with advanced solid tumors (NCT01585701) (Kumar et al., 2014).

It is important to note that pan-ROCK inhibition using Y-27632 has been shown to increase the proliferation and migration of a number of *in vitro* and *in vivo* cancer models (Adachi et al., 2011; Vishnubhotla et al., 2012; Yang and Kim, 2014; Chang et al., 2018). These effects may be explained by the observation that ROCK activation can contribute to negative feedback mechanisms that regulate pro-proliferative pathways. In a model of pancreatic cancer, EGF stimulation led to increased ROCK activation, which then negatively regulated EGFR signaling by affecting receptor trafficking. Treatment with Y-27632 removed this negative regulation and resulted in significantly increased AKT and MAPK signaling following EGF stimulation (Nakashima et al., 2011). Targeting ROCK may therefore only be appropriate in certain contexts and combination treatments may be required to avoid compensatory upregulation of other kinases involved in actomyosin function, such as LIMKs and MRCKs (Rath and Olson, 2012).

## Myotonic Dystrophy Kinase-Related CDC42-Binding Kinases (MRCKS)

In humans, myotonic dystrophy kinase-related Cdc42-binding kinases (MRCKs) form a family of three serine/threonine protein kinases that are evolutionarily related to ROCKs and play a key role in the regulation of actomyosin contractility (Zhao and Manser, 2015; Leroux et al., 2018). MRCKα and MRCKβ are ubiquitously expressed and share 85% kinase domain homology, whilst MRCKγ is more divergent and shows a more restricted tissue expression pattern (Unbekandt and Olson, 2014). All MRCK proteins have an N-terminal protein kinase domain followed by a C-terminal protein kinase C conserved region (C1), PH-like domain, citron homology (CH) domain and CRIB domain (Figure 3). Membrane localization of MRCKs may be mediated at least in part by the C1 domain, which has been shown to bind phorbol esters (Choi et al., 2008; Talman et al., 2014), and the PH-like domain, which may bind membrane lipids. The CH domain may facilitate substrate docking or engage in protein-protein interactions that specify protein localization (Unbekandt and Olson, 2014). The CRIB domains of MRCKα and MRCKβ bind GTP-loaded Cdc42 (Leung et al., 1998) and Rac1 (Ng et al., 2004; Schwarz et al., 2012), whilst the CRIB domain of MRCKγ has been shown to bind to RhoQ with higher affinity than Cdc42 and Rac1 (Ng et al., 2004). Although the molecular mechanisms underlying MRCK activation are not fully understood, Cdc42/Rac1 binding, membrane localization and the release of auto-inhibitory interactions appear to be required for MRCK signaling (Leung et al., 1998; Unbekandt and Olson, 2014; Zhao and Manser, 2015). Recent identification of an autophosphorylation site in MRCKα at Ser1003 led to the development of a phospho-site specific MRCKα antibody that can be used to assess levels of MRCKα activation and will likely prove useful for the further elucidation of MRCKα activation mechanisms (Unbekandt et al., 2018). Autophosphorylation of MRCKβ at Thr1108 can be used as a readout of MRCKβ kinase activity, although attempts to develop a suitable antibody tool have so far been unsuccessful (Unbekandt et al., 2019).

**FIGURE 6:**
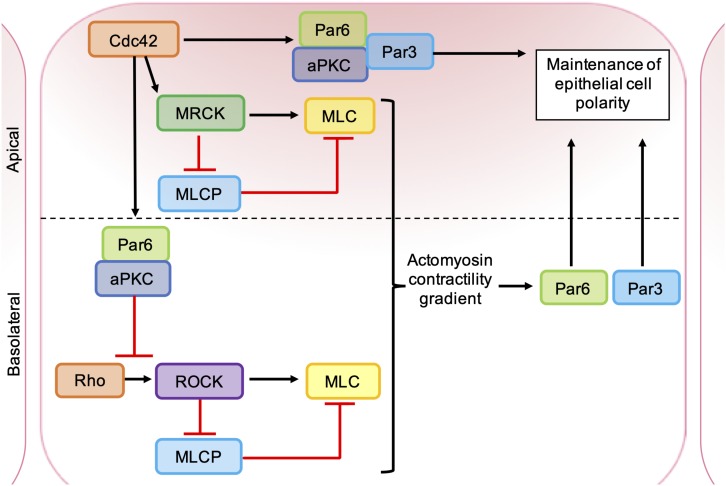
Roles of ROCK, MRCK, and aPKC in the maintenance of epithelial cell polarity. Apical MRCK activation by Cdc42 leading to a local increase in actomyosin contractility, via phosphorylation of myosin light chain (MLC) and inhibition of myosin light chain phosphatase (MLCP). Cdc42 stimulates a Par-aPKC complex, which inhibits junctional Rho-ROCK signaling and establishes an intracellular actomyosin contractility gradient, leading to the segregation of Par proteins into distinct cellular domains. Apical GTP-bound Cdc42 binds to Par6, which in turn binds aPKC and Par3. Activation of aPKC leads to the phosphorylation of key polarity proteins that maintain apical-basal cell polarity.

The catalytic domains of MRCK and ROCK are highly related and have several common substrates (Zhao and Manser, 2015). MRCKα is known to phosphorylate LIMK1/2 (Sumi et al., 2001), leading to phosphorylation and inhibition of cofilin (Ohashi et al., 2000), and also promotes MLC phosphorylation through the inhibition of MLC phosphatase (Tan et al., 2001; Wilkinson et al., 2005) and phosphorylation of MLC on Ser19 (Leung et al., 1998; Figure 5). However, ROCK and MRCK are not functionally redundant and the differential regulation of ROCK and MRCK activity plays an important role in establishing cell polarity (Figure 6). Epithelial polarization requires the segregation of PAR proteins into distinct cortical domains, which is driven at least in part by an intracellular actomyosin activity gradient. Apical actomyosin contractility is stimulated by Cdc42-MRCK activity, whilst RhoA-ROCK activity regulates contractility along cell-cell junctions. Apical Cdc42 activity leads to stimulation of a Par6-aPKC complex, which in turn inhibits junctional RhoA-ROCK signaling, establishing an actomyosin contractility gradient (Etienne-Manneville and Hall, 2001; Zihni et al., 2017). Differential regulation of ROCK and MRCK between different tumor cells may also be important for invasion. Studies of squamous cell carcinoma (SCC) cells and stromal fibroblasts in 3D co-cultures found that collective invasion required Rho-ROCK signaling in fibroblasts and Cdc42-MRCK signaling in SCC cells. Imaging revealed that fibroblasts always act as the “leading cell,” whilst SCC cells migrate through the channels in the ECM created by the fibroblast. This remodeling of the ECM by fibroblasts was dependent on both extracellular protease activity and the generation of contractile force by Rho-ROCK signaling. In contrast, the SCC cells were found to rely on Cdc42-MRCK-mediated regulation of MLC to follow leading the fibroblasts (Gaggioli et al., 2007).

MRCKα expression is increased in a number of human cancers (Unbekandt and Olson, 2014) and dual inhibition of ROCK and MRCK activity has been shown to produce a greater inhibition of cancer cell migration and invasion than blocking ROCK alone (Wilkinson et al., 2005; Kale et al., 2014). Widely used ROCK inhibitors such as Y-27632 and fasudil bind to MRCKs (Heikkila et al., 2011), although these compounds also inhibit several other kinases and are not clinically viable (Bain et al., 2007). Whilst dual ROCK/MRCK inhibition remains a valid therapeutic strategy, selective MRCK inhibitors may prove beneficial in overcoming the dose-limiting hypotension associated with long-term ROCK inhibitor treatment (Kale et al., 2015). The recently identified azaindole compounds BDP8900 and BDP9066 shown to have high selectivity for MRCKα and MRCKβ over ROCK1 and ROCK2 in *in vitro* kinase assays and were found to inhibit MRCK-mediated MLC phosphorylation more potently than ROCK-mediated MLC phosphorylation in a breast cancer cell line. MRCKα and MRCKβ are overexpressed in squamous cell carcinoma (SCC) (Nindl et al., 2006) and treatment of SCC cell lines with BDP9066 led to morphological changes and decreased bundling of filamentous actin, as well as reduced cell motility and invasion. In a mouse model of SCC, topical application of BDP9066 reduced MRCKα activation in the skin and resulted in a decrease in tumor volume relative to controls. In a screen of 757 human cancer cell lines from 40 different cancer types, BDP8900 and BDP9066 treatment produced consistent anti-proliferative effects, suggesting MRCK may be a valid therapeutic target in a number of cancers other than SCC (Unbekandt et al., 2018). It is important to note that the toxicity of systemic BDP8900 and BDP9066 administration has not been studied and information regarding the bioavailability of these compounds is not yet available. The development of these novel MRCK-selective inhibitors will nevertheless prove valuable for the further pre-clinical validation of MRCK as a therapeutic target and may inform the future development of MRCK inhibitors better suited for clinical use.

## Atypical Protein Kinase CS (APKCS)

The protein kinase C (PKC) family comprises three subfamilies of serine/threonine kinases; classic PKCs (cPKCs; PKCα, PKCβ, and PKCγ), novel PKCs (nPKC; PKCδ, PKCε, PKCη, and PKCθ) and atypical PKCs (aPKC; PKCξ and PKCι). All PKC proteins exist in a basally auto-inhibited conformation, which is mediated by an interaction between the conserved C-terminal kinase domain and an N-terminal regulatory region, which comprises a pseudosubstrate motif and other structural domains that vary between PKC subfamilies (Figure 3). Classic PKCs require diacylglycerol (DAG), phosphatidylserine (PS) and Ca^2+^ for activation, whilst nPKCs require only interaction with DAG and PS (Isakov, 2018). In contrast, aPKCs are primarily activated by protein-protein interactions mediated by an N-terminal Phox Bem1 (PB1) domain and can also be regulated by PIP_3_ as well as by specific phosphorylation events (Garg et al., 2014). Through forming a complex with Par6 and Par3, aPKCs play a key role in establishing both the apical-basal polarity of epithelial tissues and the front-to-rear polarity required for cell migration downstream of Cdc42.

Most human cancers arise from epithelial tissues and the disruption of apical-basal epithelial polarity is considered an early event in tumorigenesis (Fomicheva et al., 2019). During the establishment of epithelial cell polarity, GTP-bound Cdc42 binds to Par6 via its semi-CRIB domain, which in turn binds to PKCι via a PB1-PB1 interaction (Hirano et al., 2005). The interaction of Cdc42 with Par6-PKCι results in allosteric PKCι activation and recruitment of Par3, which binds to PKCι and Par6 via its PDZ domain. Par3 then targets the complex to the apical compartment of the cell membrane through its interaction with several cell adhesion molecules (Figure 6). Apical aPKC activity then maintains apical-basal polarity through the phosphorylation of a number of polarity proteins, including MARCKS, LGN, Lgl, GSK3β, Par1, Crumbs, and Lin5/NuMA (Chen and Zhang, 2013). One mechanism by which human tumors lose polarity is through loss of Par3, which leads to mislocalization of PKCι in the cytoplasm and nucleus. Mislocalized PKCι can remain associated with Par6 and has been shown to contribute to maintenance of the transformed phenotype (reviewed in Parker et al., 2014).

The Par6-aPKC complex also plays a crucial role in establishing the front-back polarity required for directional cell migration and invasion. PKCζ has been shown to promote invasion and metastasis of breast cancer xenografts in mice by promoting the nuclear localization of NFκB-p65 and suppressing the expression of junctional proteins E-cadherin and ZO1 (Paul et al., 2015). PKCι has also been shown to stimulate cell migration by regulating the trafficking of protease-containing vesicles to the plasma membrane of invadopodia. In invasive breast cancer cell lines, the secretion of type 1-matrix metalloproteinase (MT1-MMP) was shown to be dependent on the interaction of cortactin and dynamin-2 on MT1-MMP containing endosomes. This interaction was found to be regulated by the PKCι-mediated phosphorylation of cortactin on Ser261 and depletion of PKCι led to a decrease in the release of MT1-MMP from invadopodia and a reduction in the degradative capacity of the cells (Rosse et al., 2014).

In non-small cell lung cancer (NSCLC), PKCι has been shown to promote cell survival and invasion through its action on the Rho GEF Ect2. Binding of Ect2 to the Par6-PKCι leads to phosphorylation of Ect2 on T328, which enhances its GEF activity toward Rac1 (Justilien et al., 2011). PKCι-mediated Rac1 activation can stimulate MEK-ERK signaling in pancreatic cancer cell lines, leading to increased anchorage-independent growth *in vitro* and promoting tumor growth and metastasis in an orthotopic mouse model (Scotti et al., 2010).

Overexpression of PKCι has been observed in several human cancers, including gastric (Hashimoto et al., 2019), pancreatic (Scotti et al., 2010), breast (Rosse et al., 2014), ovarian (Tsang et al., 2017), prostate (Apostolatos et al., 2018), and lung cancer (Kim et al., 2019) and has been shown to act as an oncogenic driver in numerous studies (reviewed in Parker et al., 2014). The role of PKCξ in cancer is less clear, with both increased and decreased expression reported in human tumors (Garg et al., 2014). Targeting aPKCs in cancer may therefore only be appropriate in certain contexts. Several ATP-competitive inhibitors have been described that show selectivity for PKCξ/PKCι over cPKC and nPKC isoforms. For example, CRT0066854 potently inhibits PKCι and PKCξ, with some off-target inhibition of ROCK2 and PRK2, and was shown to inhibit anchorage-independent cell growth and migration *in vitro* (Kjaer et al., 2013). More recently, the selective aPKC inhibitors 2-acetyl-1,3-cyclopentanedione (ACPD) and 3,4-diaminonaphthalene-2,7-disulfonic acid (DNDA) were found to suppress the proliferation of melanoma cell lines showing high levels of PKCι and PKCξ expression, whilst having little effect on the proliferation of normal melanocytes (Ratnayake et al., 2017). Compounds that block protein-protein interactions at the PB1 domain may provide greater selectivity for aPKCs over other protein kinases. The gold compound aurothiomalate (ATM) selectively inhibits the binding of Par6 to PKCι and PKCξ (Erdogan et al., 2006; Stallings-Mann et al., 2006; Butler et al., 2015) and has been shown to inhibit tumor growth in animal models (Butler et al., 2015; Tsang et al., 2017). ATM is approved for the treatment of rheumatoid arthritis (RA) and a phase I study found ATM to be well tolerated in patients with advanced NSCLC, ovarian cancer and pancreatic cancer (Mansfield et al., 2013). Another gold compound used to treat RA, auranofin (ANF), is reported to block the interaction of PKCι with Par6 more potently than ATM (Parker et al., 2014) and phase I/II trials are ongoing to investigate the effect of ANF in combination with an mTOR inhibitor in lung cancer (NCT01737502).

## Concluding Remarks

The role of Rho GTPases as key regulators of cell migration and invasion has been recognized for decades, yet few compounds targeting Rho GTPase signaling networks have been developed beyond an early preclinical stage. Due to the challenges of inhibiting Rho GTPase activation directly, targeting Rho GTPase effectors remains the most promising approach. Whilst PAK and ROCK inhibitors have progressed to phase I clinical trials, further work is needed to elucidate the context-dependent roles of Rho GTPase effectors and to identify compensatory feedback networks which may limit the success of these targeted therapies. Genome sequencing of human tumors has identified several mutations in Rho GTPases, yet the functional and clinical significance of many of these mutants remain poorly understood. The generation of mouse models with tissue-specific Rho GTPase alterations will be crucial in understanding the role of these mutants in cancer and may lead to the identification of novel therapeutic targets within Rho GTPase signaling networks.

## Author Contributions

NC and AR wrote, corrected, and reviewed the final manuscript.

## Conflict of Interest

The authors declare that the research was conducted in the absence of any commercial or financial relationships that could be construed as a potential conflict of interest.
